# Integrating myocardial CT perfusion with coronary CT angiography improves risk stratification in patients with dialysis-dependent end-stage renal disease

**DOI:** 10.1007/s11604-024-01690-5

**Published:** 2024-11-02

**Authors:** Suguru Araki, Kakuya Kitagawa, Satoshi Nakamura, Florian Michallek, Takanori Kokawa, Masafumi Takafuji, Hajime Sakuma

**Affiliations:** 1 Department of Radiology, Mie University Hospital, 2-174 Edobashi, Tsu, Mie 514-8507 Japan; 2 Regional Co-creation Deployment Center, Mie Regional Plan Co-creation Organization, Mie University, 1557 Kurimamachiyacho, Tsu, Mie 514-8507 Japan; 3 Department of Advanced Diagnostic Imaging, Mie University Graduate School of Medicine, 2-174 Edobashi, Tsu, Mie 514-8507 Japan; 4 Department of Radiology, Charité-Universitätsmedizin Berlin, Charitéplatz 1, 10117 Berlin, Germany; 5 Clinical Research Support Center, Mie University Hospital, 2-174 Edobashi, Tsu, Mie 514-8507 Japan

**Keywords:** Cardiac computed tomography, Coronary computed tomography angiography, Dynamic computed tomography perfusion, Hemodialysis, End-stage renal disease

## Abstract

**Purpose:**

Risk stratification for incidence of major adverse cardiovascular events (MACE) in patients with dialysis-dependent end-stage renal disease (dd-ESRD) is challenging. Moreover, the usefulness of coronary CT angiography (CCTA) is often limited because of high calcification. This study aimed to investigate the prognostic value of comprehensive cardiac CT in patients with dd-ESRD for predicting MACE.

**Materials and methods:**

This retrospective analysis included 92 patients with dd-ESRD who underwent comprehensive cardiac CT. Obstructive coronary artery disease (CAD) was defined by CCTA with > 50% stenosis. Global myocardial blood flow (MBF) and summed stress score (SSS) were obtained through dynamic CTP. Cox regression analysis was used to assess correlation with MACE. Kaplan–Meier curves were used to estimate cumulative event rates, and the global Chi-square test was used to assess the incremental value of dynamic CTP over CCTA.

**Results:**

During a median follow-up of 2.3 years, 43 patients experienced MACE. Univariate analysis revealed that presence of obstructive CAD, higher SSS, and lower global MBF were significantly associated with increased risk of MACE. In multivariable analysis, lower global MBF and presence of obstructive CAD were independently associated with MACE (*p* = 0.02, and *p* = 0.04, respectively). CCTA and dynamic CTP combination had incremental value over CCTA alone for predicting MACE, respectively (global Chi-square score, 19.3 and 11.7, respectively).

**Conclusion:**

Presence of obstructive CAD on CCTA and lower global MBF on dynamic CTP are independently associated with increased risk of MACE in patients with dd-ESRD. The addition of dynamic CTP to CCTA may improve risk stratification in this population.

**Supplementary Information:**

The online version contains supplementary material available at 10.1007/s11604-024-01690-5.

## Introduction

Coronary artery disease (CAD) poses a significant threat to the health and well-being of patients with chronic kidney disease (CKD), including those with end-stage renal disease (ESRD), particularly when dialysis dependent (dd-ESRD). The prevalence of CAD among individuals with moderate-to-severe renal dysfunction is extremely high, and cardiovascular disease remains the leading cause of mortality in this population [1]. The age-adjusted cardiovascular mortality rate among patients with dd-ESRD is 10–20 times higher compared to the general population [2]. However, early referral for cardiac catheterization and coronary revascularization has shown potential for improving outcomes in patients with moderate to severe CKD [3].

Previous studies have identified a strong association between CKD and major adverse cardiovascular events (MACE), emphasizing the need for accurate risk stratification in this vulnerable population [4–6]. However, diagnosis of CAD in patients with dd-ESRD can be challenging owing to atypical presentations and a lower prevalence of classic symptoms [7]. Traditional risk assessment tools, such as the Framingham score, have limited prognostic power in patients with CKD and ESRD, and the increased risk of adverse cardiovascular events in these individuals cannot be completely explained by conventional risk factors [2].

Despite the high cardiovascular risk burden in patients with dd-ESRD, most cardiovascular clinical trials have excluded or underrepresented individuals with ESRD. In a large retrospective review of patients who underwent renal transplantation, nuclear stress testing demonstrated only modest sensitivity and poor specificity for the detection of CAD [8]. While one study identified coronary artery calcification as an independent predictor of death in patients undergoing hemodialysis [9], the precise role of coronary artery calcification as a prognostic indicator in patients with ESRD remains unclear [10]. Evaluating CAD using CCTA can also be difficult in this population because of the high calcification burden, although several studies have noted the effectiveness of CCTA [11].

Therefore, we aimed to explore the prognostic value of comprehensive cardiac CT, including coronary CT angiography (CCTA), dynamic stress CT perfusion (CTP), and delayed enhancement CT in predicting MACE in patients with dd-ESRD, thereby addressing the diagnostic gap in this patient population.

## Materials and methods

### Study population

**Fig. 1:**
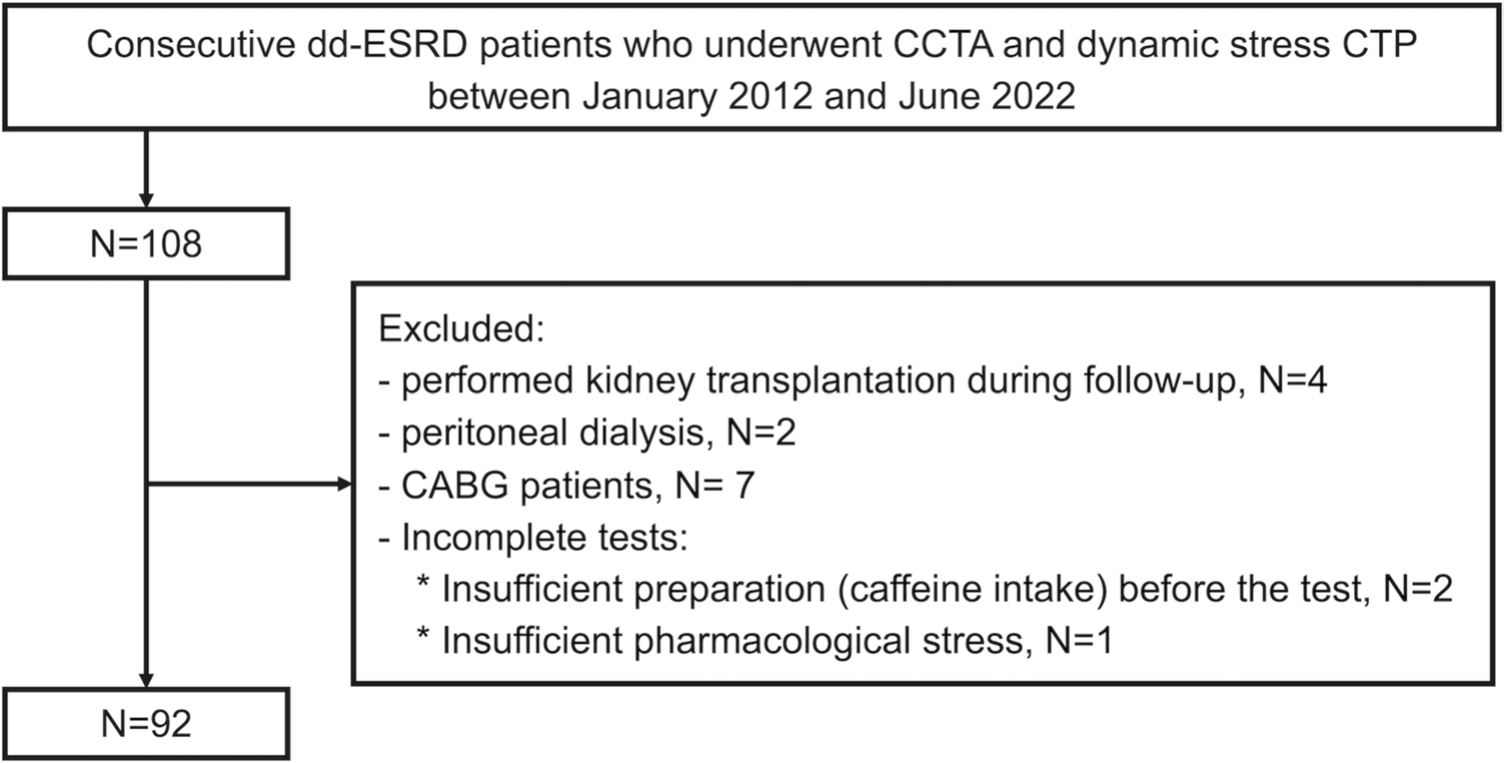
Flowchart depicting the patient selection process. *dd-ESRD* dialysis-dependent end-stage renal disease, *CCTA* coronary CT angiography, *CTP* CT perfusion, *CABG* coronary artery bypass graft

This retrospective study was approved by the local institutional review board (approval no. H2023-231). A total of 108 consecutive patients with dd-ESRD who were referred to our hospital with known or suspected CAD between January 2012 and June 2022 were considered for inclusion. The exclusion criteria were as follows: (1) incomplete tests or severe artifacts owing to motion or breathing, (2) kidney transplantation during follow-up, (3) peritoneal dialysis, and (4) history of coronary artery bypass graft (CABG). Flowchart of the patient selection process is illustrated in Fig. 1.

### Image acquisition

All patients included in the study underwent imaging using a second-generation dual-source CT scanner (Definition Flash, Siemens Healthineers) or third-generation dual-source CT scanner (Somatom Force, Siemens Healthineers). Prior to dynamic stress CTP, patients were advised to abstain from consuming caffeine for a minimum of 24 h. After anteroposterior and lateral topograms, unenhanced CT images for coronary artery calcium scoring were performed using ECG-triggered prospective high-pitch helical scan at 100 kV with an Sn filter. For the acquisition of dynamic stress CTP, patients were administered 20 mg of adenosine triphosphate at a rate of 160 mg/kg/min for more than 3 min. A contrast medium with an iodine concentration of 370 mg/ml (Iopamilon-370, Bayer AG) was injected at a flow rate of 5 ml/s and a total volume of 40 ml. Dynamic CTP were acquired over 30 s using an electrocardiography (ECG)-triggered axial scan mode at two alternating table positions with a Z-axis coverage of 73 mm or 102 mm (“shuttle mode”). Tube voltage was set to 80 kVp or 70 kVp and the tube current was determined using an automatic exposure control system, aiming for equivalent quality at a reference of 350 mA per rotation at 120 kVp. Throughout the procedure, ECG, blood pressure, and percutaneous oxygen saturation were continuously monitored and recorded. Adenosine triphosphate administration was discontinued after data acquisition. Ten minutes after acquisition of dynamic CTP data, ECG-triggered prospective axial CCTA was performed at rest with the coronary arteries dilated with a sublingual nitrate (Myocor sprays, Toa Eiyo Co., Ltd.). The scan parameters used for this phase were a tube voltage of 100 kVp, 80 kVp, or 70 kVp and a gantry rotation time of 0.28 s or 0.25 s. The contrast medium (0.84 ml/kg) was injected over 12 s. The tube current was adjusted using automatic exposure control. Seven minutes after CCTA, end-systolic (250 ms after the R wave) myocardial delayed enhancement CT was acquired by electrocardiographically triggered axial scan without additional contrast medium administration (a total of 120 ml of iodine contrast medium per patient). Tube voltage was 80 kVp and tube current was adjusted using automatic exposure control [12]. Dynamic CTP and delayed enhancement CT data were both acquired in the end-systolic phase to minimize motion artifacts, even at high heart rates, and to facilitate easier comparison between them.

### Image analysis

Dynamic CTP was analyzed using commercially available perfusion software (Syngo VPCT body, Siemens Healthineers). Myocardial blood flow (MBF) was estimated using a parametric deconvolution technique based on a two-compartment model of the intravascular and extravascular spaces. To generate voxelwise MBF maps, the maximum slope method was employed and reconstructed with a slice thickness of 3 mm and 1 mm increments [13].

In short-axis view of the MBF map, polygonal regions of interest measuring 1–2 cm^2^ were placed within each of the 16 myocardial segments, according to the American Heart Association (AHA) guidelines, omitting the apex. These regions of interest were positioned to avoid areas of delayed enhancement and maintained a minimum distance of 1 mm from the endocardial and epicardial to prevent contamination by artifacts or misregistration. Global MBF was calculated by averaging the MBF values across the 16 segments. Segmental normalized MBF value was determined by dividing each segment’s MBF value by the highest MBF value among the 16 segments. A summed stress score (SSS) was calculated by summing the scores of all segments based on a 5-point scale that utilized normalized MBF values: 0 = normal (> 0.75), 1 = mildly abnormal (≤ 0.75, > 0.675), 2 = moderately abnormal (≤ 0.675, > 0.60), 3 = severely abnormal (≤ 0.60), or 4 = absent [14]. Pharmacological stress adequacy was assessed with reference to a significant increase in heart rate (greater than 10 beats per minute), a decrease in systolic blood pressure, the presence of typical stress-induced symptoms such as chest discomfort or shortness of breath during ATP infusion, and whether the global stress MBF value showed a sufficient rise. If these criteria were not met, the pharmacological stress was considered insufficient [15].

To assess CAD on CCTA, a joint reading was performed by at least two observers, including a radiologist with more than 10 years of experience in CCTA. Coronary segments with a reference diameter of ≥ 1.5 mm were evaluated for the detection of stenosis. The severity of CAD on CCTA was graded using the Coronary Artery Disease-Reporting and Data System (CAD-RADS) [16]. Obstructive CAD was defined as ≥ 50% stenosis in ≥ 1 vessel (CAD-RADS ≥ 3).

The presence or absence of delayed enhancement was determined visually. Delayed enhancement was classified into ischemic (infarction) and non-ischemic patterns. An ischemic pattern of delayed enhancement is characterized by subendocardial to transmural enhancement that follows the distribution of the coronary arteries. All other patterns are categorized as non-ischemic [17].

### Follow-up

Follow-up information was obtained by conducting a comprehensive review of hospital records or telephone interview. Telephone interviews were conducted with patients who had not visited our outpatient department within the past 3 months. MACE included cardiac death, nonfatal myocardial infarction (MI), unstable angina, hospitalization owing to congestive heart failure, and late revascularization (occurring > 90 days after examination) [18].

Cardiac death was defined as fatal outcome owing to acute MI, ventricular arrhythmia, and congestive heart failure. Nonfatal MI was defined as prolonged angina accompanied by new electrocardiographic abnormalities and elevated cardiac biomarker levels. Unstable angina was defined as a new-onset or worsening angina, including angina occurring at rest that necessitated hospital admission. Congestive heart failure was determined by the emergence of relevant symptoms (e.g., cough, shortness of breath, dyspnea on exertion, paroxysmal nocturnal dyspnea, and reduced exercise tolerance) in conjunction with either new radiological, echocardiographic, or biochemical findings consistent with congestive heart failure or the presence of physical signs, including pulmonary rales, S3 gallop sounds, and weight gain.

### Statistical analysis

Statistical analysis was carried out using JMP14.2.0 (SAS, Institute Cary, NC, USA) software. Continuous data are expressed as medians with interquartile range (IQR) or counts with percentages. To detect independent predictors associated with MACE, we compared each parameter between the groups using Cox regression analysis. Only variables with *p* < 0.10 on univariate analysis were included in the multivariate analysis to avoid overfitting. We constructed multivariable models to address potential collinearity by including global MBF and SSS obtained from dynamic CTP. Incremental values of CCTA over traditional coronary risk factors and CTP over CCTA, were evaluated by the global chi-square test. Kaplan–Meier curves were used to estimate the cumulative event rates for CTP and CCTA. In all tests, statistical significance was defined as *p* < 0.05 after Bonferroni correction, where applicable.

## Results

### Patient characteristics

In total, 108 patients met the inclusion criteria, of which 16 were excluded [underwent kidney transplantation during follow-up, *n* = 4; peritoneal dialysis, *n* = 2; history of CABG, *n* = 7; insufficient preparation (caffeine intake) before the test, *n* = 2; and insufficient pharmacological stress, *n* = 1]. A flowchart of the study is presented in Fig. 1. Patient characteristics are presented in Table 1. The final study population consisted of 92 patients (*n* = 68 male, 73.9%). The median age was 67 years (IQR 58–74 years), median height was 163 cm (IQR 157–169 cm), median body weight was 60 kg (IQR 53–71 kg), and median dialysis vintage was 4.4 years (IQR 1.6–8.6 years). The dose length products for CCTA and CTP were 215 (IQR 129–324) mGy cm and 282 (IQR: 231–339) mGy cm, respectively, and the effective dose for the two techniques were 3.0 (IQR 1.8–4.5) mSv and 3.9 (IQR 3.2–4.7) mSv, respectively, using a conversion coefficient of 0.014.

**Table 1:** Baseline patient characteristics

	*N* = 92
Age (years)	67 (58–74)
Male, *n* (%)	68 (73.9)
Height (cm)	163 (157–169)
Body weight (kg)	60 (53–71)
Dialysis vintage (years)	4.4 (1.6–8.6)
Coronary risk factors, *n* (%)	
Hypertension	84 (91.3)
Diabetes	55 (59.7)
Dyslipidemia	43 (46.7)
Current smoker	58 (63.0)
Family history of CAD	14 (15.2)
History of revascularization	35 (38.0)
Indication for CT examination, *n* (%)	
Exertional chest pain	39 (42.3)
Echocardiographic abnormality	14 (15.2)
ECG abnormality	8 (8.7)
Evaluation of coronary stent	19 (20.6)
Chest pain at rest	4 (4.3)
Others	8 (8.9)

Data are presented as the median (interquartile range) or number of patients (%)

*CAD* coronary artery disease, *ECG* electrocardiography

### Imaging results

**Fig. 2:**
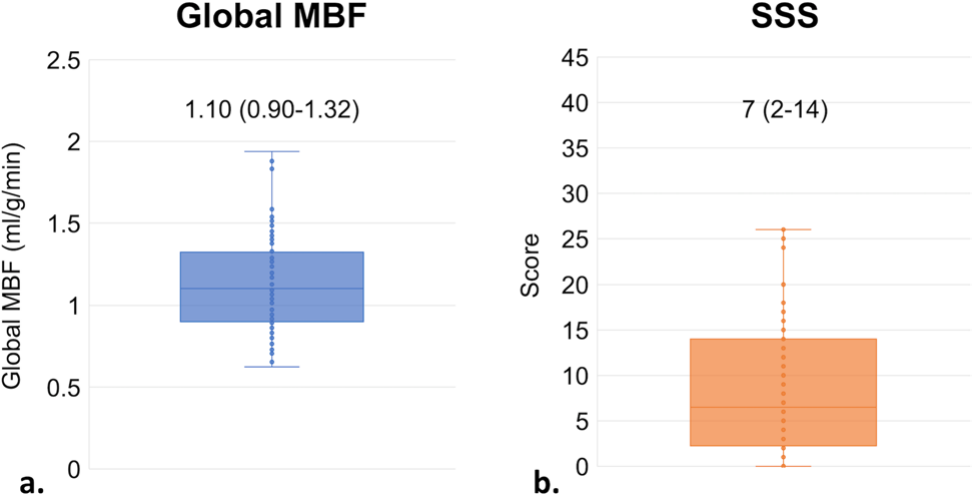
The distribution of global myocardial blood flow (**a**) and summed stress scores (**b**) of the cohort

The results of the CCTA, dynamic CTP, and delayed enhancement are presented in Table 2 and Fig. 2. CAD-RADS scores of 0, 1, 2, 3, 4A, 4B, 5 was observed in 8 (8.6%), 3 (3.2%), 15 (16.4%), 13 (14.1%), 35 (38.2%), 6 (6.5%), 12 (13.0%) patients, respectively. In patients with CAD-RADS 3 or above, 47 vessels (17.0%) in 34 patients (37.0%) were assessed as non-diagnostic (modifier N), while there were no patients classified as a CAD-RADS category N (non-diagnostic study). Obstructive CAD was detected in 66 (71.7%) patients. The median global MBF value was 1.10 ml/g/min (IQR 0.90–1.32 ml/g/min). Median SSS value was 7 (IQR 2–14). Delayed enhancement was observed in 39 (42.4%) patients, and ischemic and non-ischemic patterns were observed in 34 (37.0%) and 5 (5.4%) patients, respectively.

**Table 2:** Imaging results

Ca score in patients without coronary stent	1322 (237–3335)
Obstructive CAD, *n* (%)	
None	26 (28.3)
1-Vessel disease	23 (25.0)
2-Vessel disease	25 (27.2)
3-Vessel disease	18 (19.5)
CAD-RADS, *n* (%)	
0	8 (8.6)
1	3 (3.2)
2	15 (16.4)
3	13 (14.1)
4A	35 (38.2)
4B	6 (6.5)
5	12 (13.0)
Delayed enhancement	
None	53 (57.6)
Ischemic pattern	34 (37.0)
Non-ischemic pattern	5 (5.4)

*CAD* coronary artery disease, *RADS* Reporting and Data System

### Outcomes

In a median follow-up period of 2.3 years, (range 0.3–9.1 years), 43 patients (46.7%) experienced MACE, including cardiac death (*n* = 9), nonfatal MI (*n* = 5), unstable angina (*n* = 1), hospitalization for exacerbation of congestive heart failure (*n* = 8), and late (> 90 days) revascularization (*n* = 20). Non-cardiac death occurred in seven patients.

**Fig. 3:**
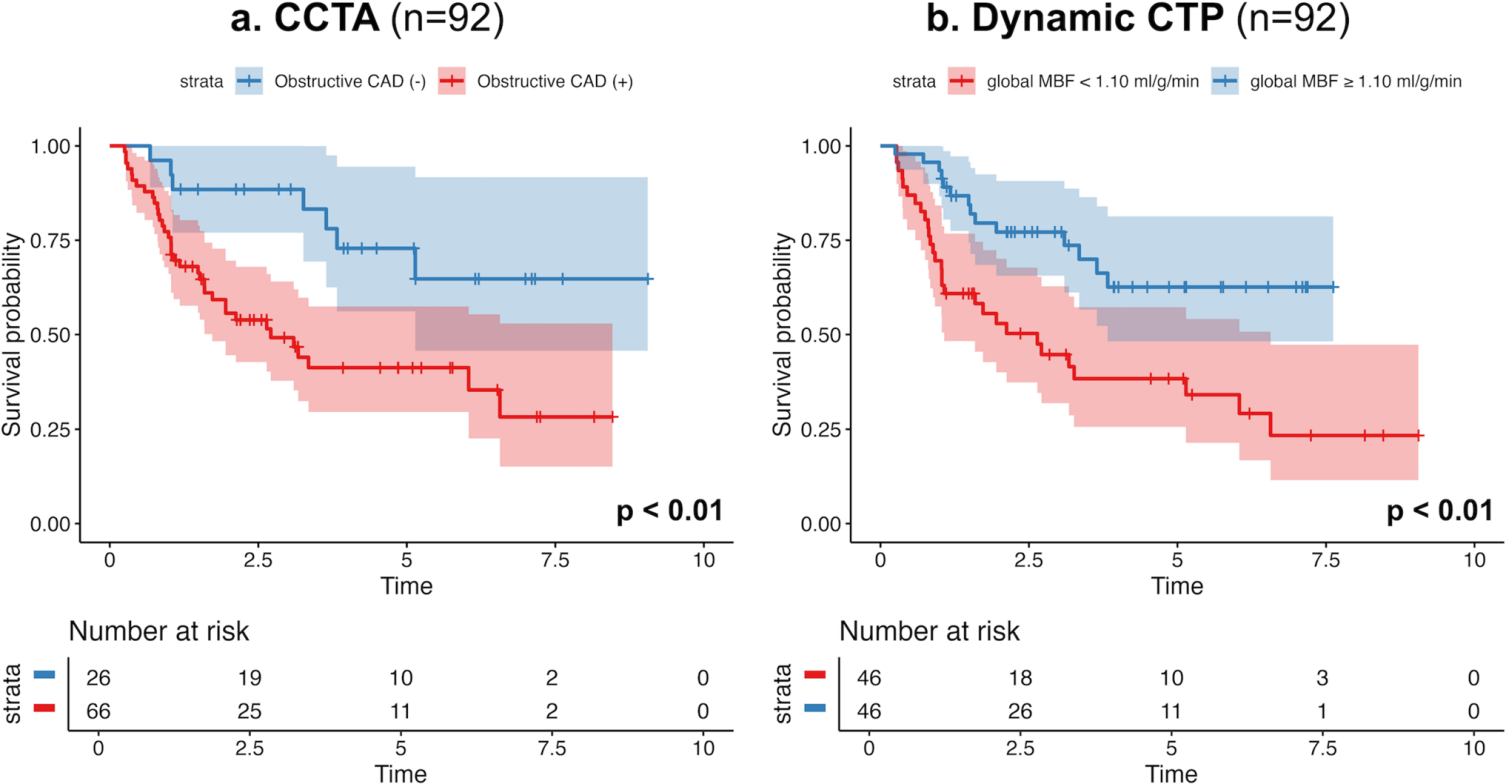
Kaplan–Meier curves in patients with and without obstructive coronary artery disease by coronary CT angiography (**a**) and in patients with global myocardial blood flow below and above median (1.10 ml/g/min) by dynamic CT perfusion (**b**) for all events

**Fig. 4:**
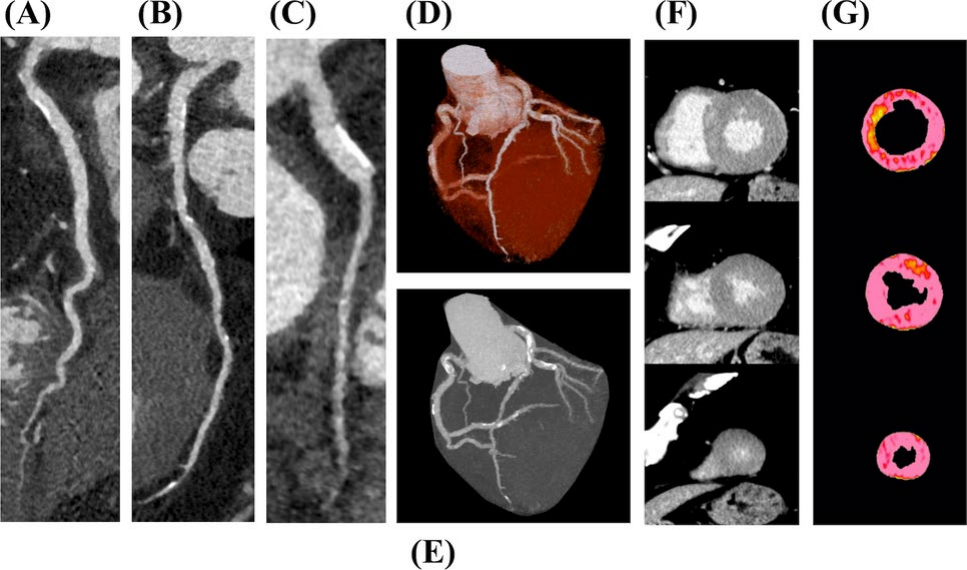
Cardiac CT images of a 70-year-old male who had not experienced MACE during a follow-up period of 5.1 years. **A** Curved multiplanar reconstruction of the right coronary artery, **B** curved multiplanar reconstruction of the left anterior descending coronary artery, **C** curved multiplanar reconstruction of the left circumflex coronary artery, of which CAD-RADS score was 2, indicating the absence of obstructive CAD with calcium score of 869, **D** volume rendering image of the heart and coronary arteries, **E**: maximum intensity projection image of the heart and coronary arteries, **F** delayed enhancement images from base, mid, and apex, and **G** MBF maps from base, mid, and apex. The global MBF of the patient was 150.0 ml/g/min; *MACE* major adverse cardiac event, *CAD-RADS* Coronary Artery Disease-Reporting and Data System, *CAD* coronary artery disease, *MBF* myocardial blood flow

**Fig. 5:**
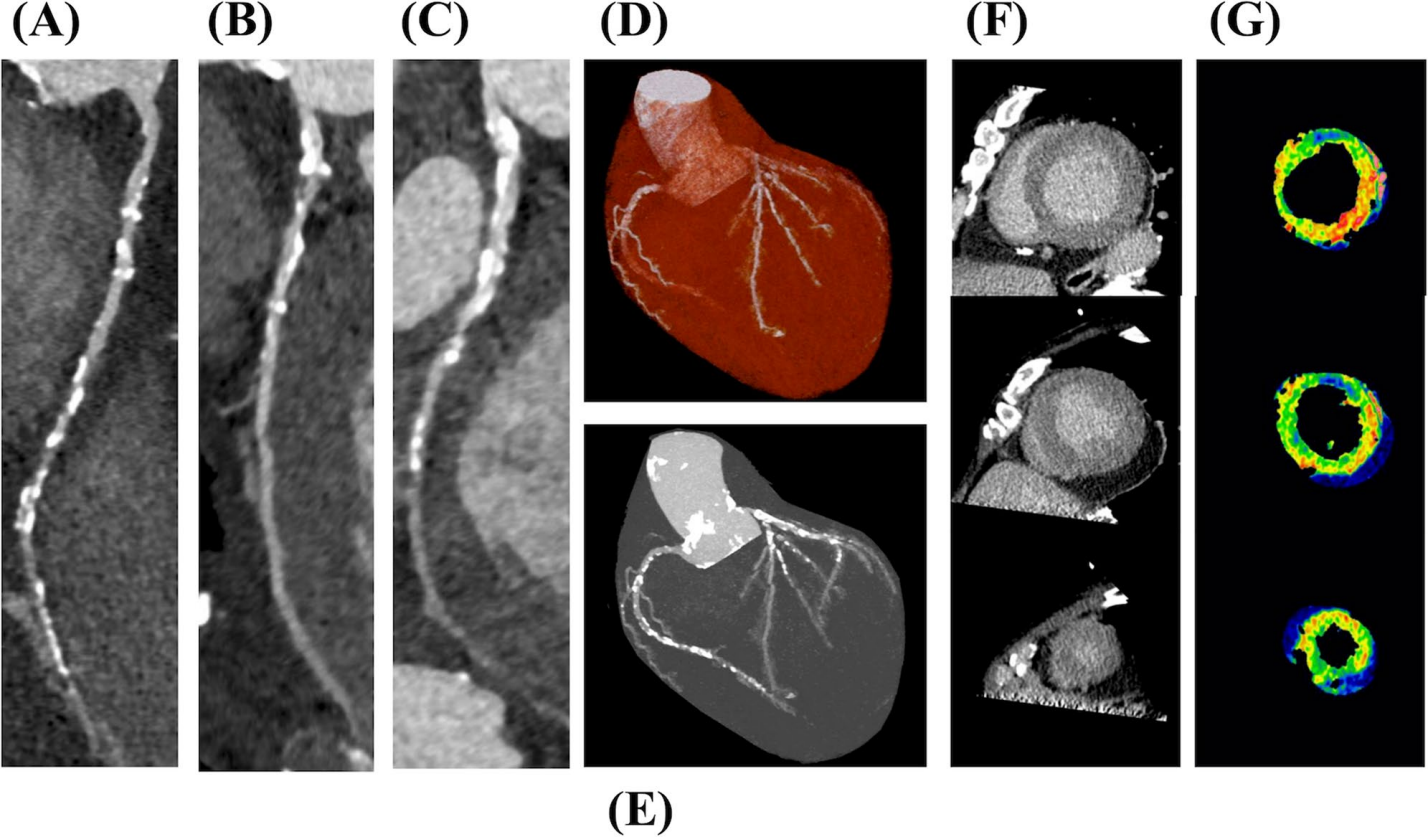
Cardiac CT images of a 70-year-old male who experienced a major adverse cardiac event (MACE). Eleven months after the examination, the patient was hospitalized due to heart failure exacerbation. **A** Curved multiplanar reconstruction of right coronary artery, **B** curved multiplanar reconstruction of the left anterior descending coronary artery, **C** curved multiplanar reconstruction of the left circumflex coronary artery. Multiple moderate to severe stenoses are present in each vessel, with a CAD-RADS score of 4A, indicating obstructive CAD and a calcium score of 3340, **D** volume rendering image of the heart and coronary arteries, **E** maximum intensity projection image of the heart and coronary arteries, **F** delayed enhancement images from the base, mid, and apex, and **G** MBF maps from base, mid, and apex. The global MBF of the patient was 80.1 ml/g/min; *MACE* major adverse cardiac event, *CAD-RADS* Coronary Artery Disease-Reporting and Data System, *CAD* coronary artery disease, *MBF* myocardial blood flow

Univariate and multivariate Cox regression analyses were performed to assess factors affecting the incidence of MACE. Among the parameters that can be obtained from comprehensive cardiac CT, the calcium score was excluded from the regression analysis because some patients in our cohort have implanted coronary stents, making it impossible to measure the calcium score in all patients. In univariate analysis, lower global MBF (< 1.10 ml/g/min), higher SSS (≥ 7), and presence of obstructive CAD were significantly associated with MACE [hazard ratio (HR) 2.69; 95% confidence interval (CI) 1.42–5.10; *p* < 0.01, HR 1.97; 95% CI 1.06–3.67; *p* = 0.03, HR 3.09; 95% CI 1.36–6.98; *p* < 0.01, respectively] (Table 3). These characteristics were subsequently included in the multivariable analysis to construct two models: Model 1 was composed of global MBF and the presence of obstructive CAD, while Model 2 was composed of SSS and obstructive CAD, because global MBF and SSS are possible confounding factors. In Model 1, lower global MBF and the presence of obstructive CAD retained their significance, respectively (HR 2.20.; 95% CI 1.14–4.25; *p* = 0.02; HR 2.44; 95% CI 1.06–5.63; *p* = 0.04), whereas higher SSS did not retain its significance in Model 2 (Table 4). The Kaplan–Meier curves for obstructive CAD and global MBF are illustrated in Fig. 3. Each factor was independently correlated with the incidence rate of MACE (*p* < 0.01). The Kaplan–Meier curves for calcium score and delayed enhancement did not show significant difference in the incidence of MACE between the groups (shown in Supplementary Fig. 1). Representative cases with and without MACE are demonstrated in Figs. 4 and 5.

**Table 3:** Univariate predictors of MACE

	Univariate analysis		
	Cutoff	HR (95%CI)	*p* value
Patient characteristics			
Male		0.84 (0.42–1.71)	0.64
Age	> 65	1.37 (0.74–2.56)	0.32
BMI	> 25	1.19 (0.63–2.26)	0.59
Dialysis vintage (per 1 year)		0.99 (0.95–1.02)	0.65
Hypertension		1.65 (0.51–5.35)	0.41
Diabetes		1.06 (0.57–1.95)	0.86
Dyslipidemia		1.32 (0.73–2.41)	0.36
Current smoker		1.42 (0.75–2.69)	0.28
Family history of CAD		1.53 (0.73–3.22)	0.26
History of revascularization		1.67 (0.91–3.09)	0.12
CT findings			
Global MBF (ml/g/min)	< 1.10	2.69 (1.42–5.10)	< 0.01
SSS	> 7	1.97 (1.06–3.67)	0.03
Obstructive CAD	(+)	3.09 (1.36–6.98)	< 0.01
Delayed enhancement	(+)	1.56 (0.86–2.86)	0.15

*MACEs* major adverse cardiac events, *HR* hazard ratio, *CI* confidence interval, *BMI* body mass index, *CAD* coronary artery disease, *MBF* myocardial blood flow, *SSS* summed stress score

**Table 4:** Multivariable Cox regression analysis

	Cutoff	Multivariable analysis			
		Model 1		Model 2	
		HR (95%CI)	p value	HR (95%CI)	p value
Global MBF (ml/g/min)	< 1.10	2.20 (1.14–4.25)	0.02		
SSS	> 7			1.56 (0.82–2.95)	0.17
Obstructive CAD	(+)	2.44 (1.06–5.63)	0.04	2.68 (1.15–6.22)	0.02

*HR* hazard ratio, *CI* confidence interval, *BMI* body mass index, *MBF* myocardial blood flow, *SSS* summed stress score, *CAD* coronary artery disease

**Fig. 6:**
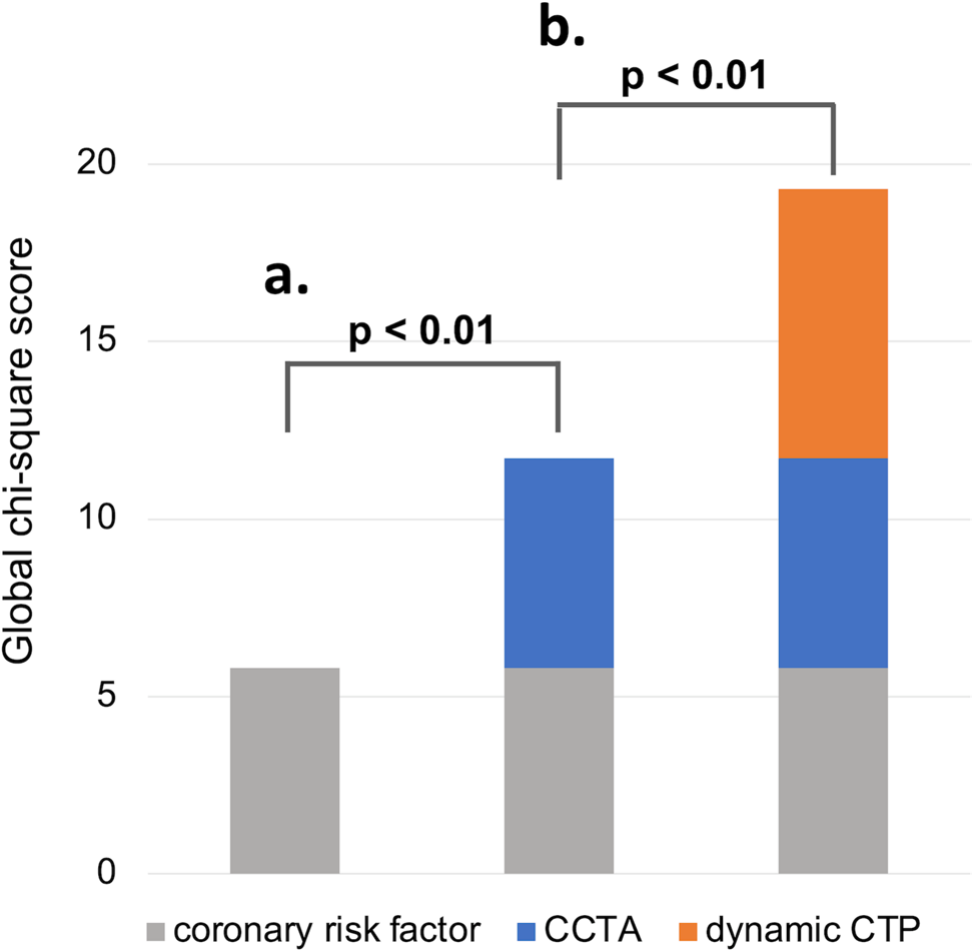
This illustration shows the incremental prognostic value of the presence of obstructive coronary artery disease by coronary CT angiography when added to coronary risk factor (a) and the incremental value of reduced global myocardial blood flow (below median of 1.10 ml/g/min) by dynamic CT perfusion added to CCTA and coronary risk factor. (b) Coronary risk factors include age, sex, smoking, family history of coronary artery disease, hypertension, dyslipidemia, and diabetes

Global Chi-square scores were calculated to assess the incremental prognostic value of CCTA and dynamic CTP (Fig. 6). The addition of obstructive CAD on CCTA to traditional coronary risk factors including age, sex, smoking, family history of CAD, hypertension, dyslipidemia, and diabetes (global Chi-square: 5.8) significantly increased the global Chi-square score (11.7; *p* = 0.008). Adding reduced global MBF to this model (global Chi-square score: 11.7) resulted in a significant increase in the global Chi-square score (19.3; *p* = 0.006).

## Discussion

We performed a retrospective study to identify the prognostic factors for patients with dd-ESRD using comprehensive cardiac CT. The following observations were made: (1) the presence of obstructive CAD on CCTA and lower global MBF on dynamic CTP were independent predictors of MACE in patients with dd-ESRD and (2) the addition of dynamic CTP to CCTA may improve risk stratification in patients with dd-ESRD. To our knowledge, this is the first study to evaluate the prognostic value of MBF quantified using dynamic CTP in patients with dd-ESRD.

As previously reported, traditional risk factors only partially elucidate the increased risk of coronary events in patients with ESRD, and the prognostic power of traditional risk prediction tools, such as the Framingham score, is limited [2]. Similarly, in the present study, traditional risk factors, such as age, sex, smoking, family history of CAD, hypertension, dyslipidemia, and diabetes were not significantly correlated with MACE. In fact, it has been suggested that prognostic prediction based solely on traditional coronary risk factors is challenging [19].

The potential of CCTA as a noninvasive test for CAD has been well described [19]. Its high negative predictive value makes it reliable for ruling out significant CAD in symptomatic patients with low to intermediate pre-test the probabilities of CAD in the general population [20]. However, patients with ESRD are often excluded from large trials [21, 22], precluding generalization to patients with ESRD, and only a few studies have investigated the diagnostic performance and prognostic value of CCTA. De Bie et al. showed that in 70 patients with dd-ESRD, the incidence of MI and revascularization on CCTA was 36% in patients with stenosis (*n* = 30) compared with 0% in patients without stenosis (*n* = 40) during a 2-year follow-up period, despite high average calcium scores [11]. Abe et al. also showed a significant difference in the incidence of cardiovascular events at 2 years between patients with and without CAD using CCTA (36% vs. 0%) [23]. Winther et al. reported that CCTA seemed superior for risk stratification of MACEs in patients with ESRD compared to traditional coronary risk factors and other cardiac imaging modalities, including single-photon emission computed tomography and invasive coronary angiography [18]. Similarly, our results also demonstrated that the presence of CAD on CCTA was a significant predictor of MACEs, which is consistent with the findings of previous studies. However, the high calcification burden, frequently observed in this population, often makes it difficult to assess degree of coronary stenosis, as heavily calcified plaques result in blooming artefacts. In this study, 37% of patients had non-diagnostic segments on CCTA.

Regarding myocardial perfusion, prior investigations have suggested that renal disease is associated with abnormal coronary vasodilator function [24], which may arise from multiple mechanisms [25], including decreased capillary density [26], leading to microvascular dysfunction and vascular remodeling in the epicardial arteries [27]. Recent positron emission tomography (PET) studies have emphasized the value of global stress MBF for evaluating prognosis in patients with CAD in the general population [28, 29]. The severity of coronary vascular dysfunction, as assessed by PET, is also an independent predictor of cardiac death in patients with moderate or severe renal impairment [30]. Thus, increasing evidence supports the accuracy of MBF quantification using dynamic CTP compared with PET [31–33]. In the present study, we demonstrated that global MBF, obtained through dynamic CTP, is also an independent predictor of MACEs in patients with dd-ESRD. This suggests that the addition of dynamic CTP to CCTA may allow for improved risk stratification of patients with dd-ESRD, which is consistent with the results of previous PET studies.

Although patients with dd-ESRD are inherently at high risk for MACE, comprehensive cardiac CT can further identify those within this group who are at even greater risk. This stratification is essential, as it allows clinicians to tailor management and follow-up strategies more effectively. Moreover, morphological evaluation of the coronary arteries alone may be insufficient, as patients with dd-ESRD typically have a severe calcium burden which can impair the diagnostic performance. Therefore, functional assessment becomes crucial. In this context, stress dynamic CTP provides a quantitative assessment of myocardial perfusion, enabling the monitoring of changes over time with relatively less invasiveness and offering essential functional information that complements morphological data.

Nonetheless, this study had some limitations. First, the study cohort was relatively small and derived from a single center, which may limit the generalizability of our findings to broader populations. Second, the patients in our study cohorts had a relatively high calcified plaque burden, which might lead to overestimation of coronary artery stenosis. Third, catheterization is lacking as an invasive reference standard. Fourth, although the total radiation dose (10.5 mSv) applied in this study was relatively small, combining CTP with CCTA inevitably increased the ionizing radiation dose, as well as the contrast medium volume, compared with CCTA alone. Fifth, this study had a relatively high number of censoring events occurring before the median survival time in the Kaplan–Meier analysis. While this does not invalidate the results, it could potentially influence the robustness of the survival curve and its ability to fully represent the true survival distribution. Despite these limitations, this is the first study to demonstrate the prognostic value of CTP in patients with dd-ESRD, which may improve the management of dd-ESRD. In conclusion, reduced global MBF is an independent predictor of MACE in patients with dd-ESRD. The presence of obstructive CAD on CCTA and a reduced global MBF on CTP were associated with an increased risk of MACE. Incorporating dynamic CTP into CCTA may enhance risk stratification in patients with dd-ESRD.

## Supplementary Information

Below is the link to the electronic supplementary material.

Supplementary file1 (PPTX 75987 KB)

## Data Availability

Derived data supporting the findings of this study are available from the corresponding author [K.K.] on request.

## Abbreviations

CAD: Coronary artery disease
CKD: Chronic kidney disease
ESRD: End-stage renal disease
dd-ESRD: Dialysis-dependent ESRD
CCTA: Coronary CT angiography
CTP: CT perfusion
CABG: Coronary artery bypass graft
ECG: Electrocardiography
MBF: Myocardial blood flow
SSS: Summed stress score
CAD-RADS: Coronary Artery Disease-Reporting and Data System
MACE: Major adverse cardiac event
MI: Myocardial infarction
IQR: Interquartile range
PET: Positron emission tomography
