# Supplementary figures and images for: Integrating myocardial CT perfusion with coronary CT angiography improves risk stratification in patients with dialysis-dependent end-stage renal disease
Source: Jpn J Radiol. 2024 Nov 2;43(3):402–11. doi: 10.1007/s11604-024-01690-5 (PMC11868328; doi:10.1007/s11604-024-01690-5)

## Slide 1
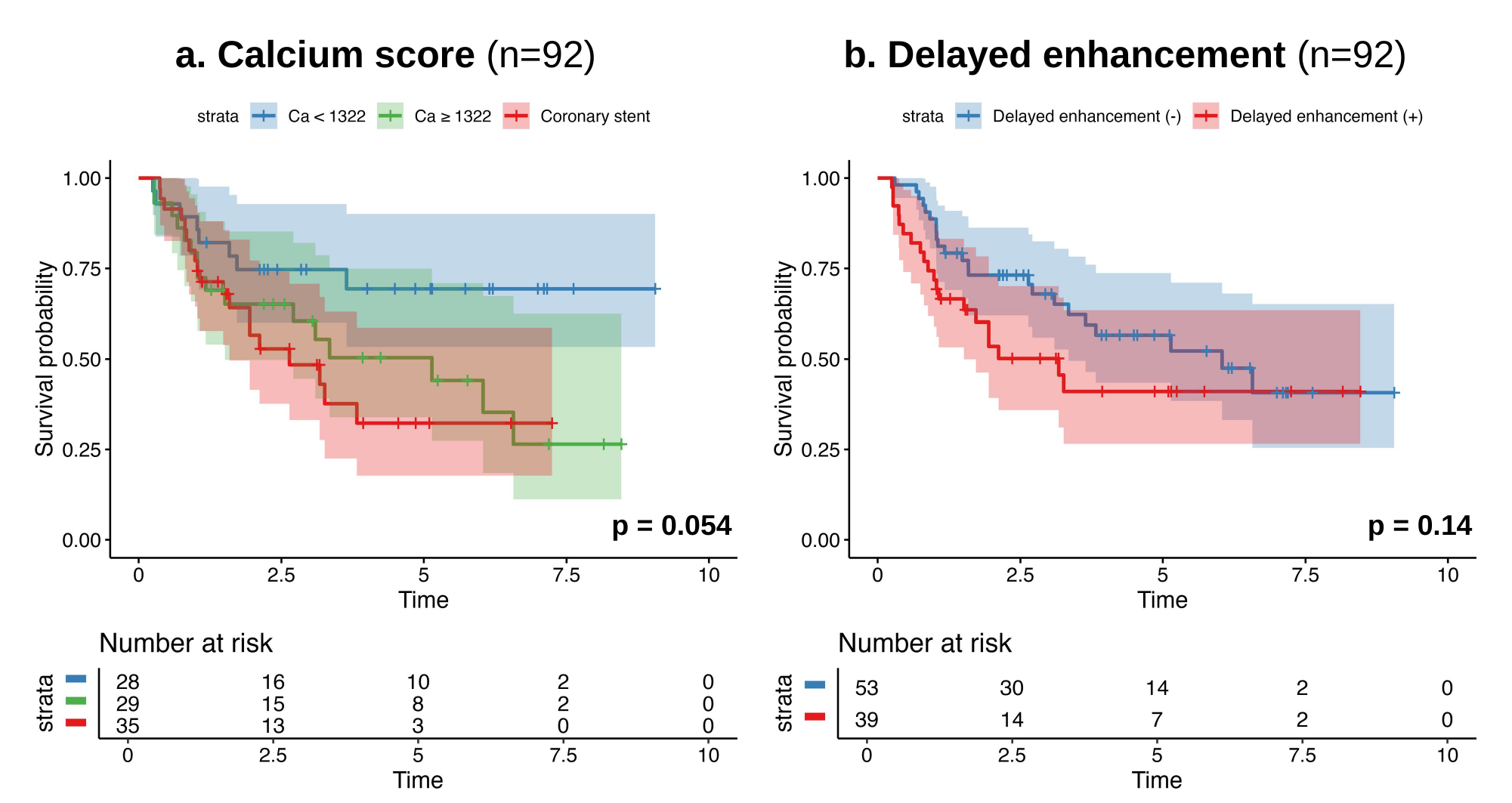

a. Calcium score (n=92)
b. Delayed enhancement (n=92)
p = 0.054
p = 0.14

Supplement: Supplementary file 1 — Supplementary file1 (PPTX 75987 KB) [file 11604_2024_1690_MOESM1_ESM.pptx]
